# Incubation temperature and parental identity determine sex in the Australian agamid lizard *Ctenophorus pictus*

**DOI:** 10.1002/ece3.4466

**Published:** 2018-09-03

**Authors:** Alexander Hansson, Mats Olsson

**Affiliations:** ^1^ Department of Biological and Environmental Sciences University of Gothenburg Gothenburg Sweden

**Keywords:** agamid lizard, *Ctenophorus pictus*, sex allocation, sex ratio, temperature‐dependent sex determination

## Abstract

Sex determination in Australian agamid lizards shows a complex framework of different mechanisms, varying even among closely related taxa. It is clear that discrete classification of these species as either having genetic (GSD) or environmental sex determination (ESD) does not agree with empirical data. Although many species in this group show temperature‐dependent sex determination (TSD), recent evidence suggests additional genetic or epigenetic effects. A proposed model explaining the adaptive significance and evolution of TSD in short‐lived agamids predicts that selection will favor temperature‐biased sex ratios in species with intense male‐male competition. Here, we use experimental incubation at (near) constant temperatures to test whether the sex of Australian painted dragons (*Ctenophorus pictus*) is influenced by temperature, building on previous research yet to have reached an agreement regarding the role of temperature in this species. In this study, incubation temperature and parental identity affected hatchling sex suggesting that environment and genetics may work in concert to determine sex in this species. Although our results are consistent with TSD, our data cannot rule out a temperature‐by‐sex effect on egg or hatchling mortality. However, our findings together with the observed differences of sex determination systems in closely related species within this genus may provide novel opportunities to address fundamental questions in the evolution of sex determination systems.

## 1 INTRODUCTION

The fundamental biological process of sex determination has been under intense scientific research for decades, mainly because of its profound implications on the phenotype of individuals and the formation of sex ratios in natural populations (West, Reece, & Sheldon, 2002). It is therefore a process of major evolutionary significance. The mechanisms of sex determination have, for a long time, been classified discretely as either genetic (GSD) or environmental (ESD). However, researchers are now in agreement that genotypic and environmental sex determination does not necessarily constitute a dichotomy, but rather two extremes of a continuum (Sarre, Georges, & Quinn, 2004).

Sex allocation studies on birds and mammals are numerous (reviewed in Wapstra et al., 2007) and show that the majority of these species have genetic sex determination. Reptiles (squamates specifically), however, show a much more diverse array of sex determination processes (Sarre et al., 2004; Shine, Elphick, & Donnellan, 2002; Wapstra et al., 2007), making them of particular interest in studies of sex allocation. The most studied environmental effect on sex determination in reptiles is temperature (temperature‐dependent sex determination [TSD]), where offspring sex is determined by the thermal regimes experienced by the embryo in the egg. TSD has been shown in all major reptile groups (Janzen & Krenz, 2004), including some squamates, most turtles, the tuatara, and all crocodilians. The widespread occurrence of TSD in reptile taxa possessing substantially different life‐histories makes the search of a single “fits all” model, explaining the adaptive significance of ESD in all reptiles, futile (Bull & Charnov, 1989).

Numerous models have been proposed to explain the evolution and adaptive significance of TSD (see reviews by Bull, 1983; Shine, 1999; Uller, Pen, Wapstra, Beukeboom, & Komdeur, 2007). However, only one model has received relatively widespread acceptance, the Charnov‐Bull model (Charnov & Bull, 1977). This model predicts that TSD will be favored over GSD when some environmental condition affects the fitness of sons and daughters differently and that neither the offspring nor the mother possesses any major predictive power over these effects. This model assumes direct fitness benefits of varying environmental regimes in the offspring and that these effects vary with sex. The model does not, however, distinguish between a direct effect on offspring fitness and an effect correlated with the actual causal factor (Warner, Uller, & Shine, 2009), nor does it recognize indirect fitness effects. These limitations may make it difficult to apply the model to predict the sex determination system of species based on their life‐history, especially in cases where temperature indirectly affects the fitness of males and females differently.

The adaptive significance of TSD in short‐lived lizards has been suggested by Warner et al. (2009) to follow a framework based on fitness effects of the timing of hatching. In contrast to the Charnov‐Bull model, this model does not attempt to explain the evolution of ESD in all reptiles, but focuses on a single family (Agamidae) containing many short‐lived lizards. This model shows that in species with intense male‐male competition for mating opportunities, newly hatched sons are likely to achieve little reproductive success in their first year when faced with competition from males of previous cohorts. The reproductive success of daughters will, however, most likely be enhanced if they hatch early enough in the season to reach sexual maturity within their first year of life (Harlow & Taylor, 2000). In this case, the fitness benefits of TSD are not direct, but a product of sex‐dependent probabilities of juveniles breeding in their first year. The model suggested by Warner et al. (2009) predicts that selection for TSD will be strongest in short‐lived reptiles with (a) a prolonged breeding season; (b) a short lifespan and early maturation; (c) intense male‐male competition; and (c) timing of hatching having little effect on juvenile survival. The Australian dragon lizards (Agamidae) are of particular interest when testing the validity of this model, as these lizards have distinct variation in life‐histories, social systems, and sex determination processes (Harlow, 2004). They have shown a complex diversity of species with GSD and TSD, with even closely related species differing in sex determining mechanisms (Harlow, 2004; Harlow & Taylor, 2000; Uller, Odierna, & Olsson, 2008). Furthermore, experimental evidence for TSD in one agamid lizard, the Australian painted dragon (*Ctenophorus pictus*), may not be consistent at reassessment of these effects (Harlow, 2004; Uller, Mott, Odierna, & Olsson, 2006). This suggests that the sex determination mechanism may differ among populations or that the inconsistencies may be due to weakness in experimental design. In the same species, female identity has consistently been shown to influence sex ratios (Uller et al., 2006), which seems to concur with the notion that something more than temperature or genes alone influences sex determination.

Here, we use Australian painted dragons to further examine the effect of incubation temperature on the sex of hatchlings, building on the work of Harlow (2004) and Uller et al. (2006), while testing the validity of the model proposed by Warner et al. (2009). Although we do not test the adaptive significance of TSD, models may be very important when attempting to predict the sex determination system of a species. The contradictory results on TSD in this species shown in Harlow (2004) and Uller et al. (2006) suggest a more dynamic sex determination system than previously discussed, confirmed by a consistent influence of female identity on sex ratios (Uller et al., 2006). Furthermore, the life‐history of *C. pictus* is in congruence with the traits listed by Warner et al.'s model, thus, predicting that this species has a temperature‐influenced sex determination system.

## 2 MATERIALS AND METHODS

### 2.1 Study species and husbandry

The Australian painted dragon is a small (8–16 g, 65–95 mm snout‐vent length [SVL]) agamid lizard occurring in open sandy habitats and low vegetation, with a habitat range from central and western New South Wales to Western Australia. They are short‐lived (only about 10% living to a second hibernation), and males are highly territorial and compete for mating opportunities (Olsson, Schwartz, Uller, & Healey, 2007). It is a sexually dimorphic species, with males being larger and polymorphic with respect to head color, typically occurring in three different morphs (red, orange, and yellow). Adult painted dragons used in this study were caught by noose or hand at Yathong Nature Reserve, New South Wales, Australia (145°35′E; 32°35′S) in October, 2014 and transported in cloth bags to holding facilities at the University of Sydney where they were housed until export to Sweden (2016) on export and import permits from Australian and Swedish authorities. The lizards were transported by airplane during hibernation. Lizards were housed in mating pairs in cages (50 L × 40 W × 35 H cm) with a sand substrate and a 40‐W spotlight at one end and rocks to allow thermoregulation and shelter use. Additional sand was added to the corner of each cage and periodically moistened to attract oviposition. The continuous presence of males is required for successful fertilization of eggs (Uller & Olsson, 2005), and males were therefore kept with females throughout the entire experiment. Males and females were assigned to mating pairs which were sustained throughout the entire experiment. All cages were sprayed daily with a mist of water and lizards were fed a variation of crickets and meal worms dusted daily with calcium and weekly with multivitamins. As thermal profiles of nests in situ have not been examined in this species, we used local ambient temperature data from the catch site in order to analyze the results in the context of seasonal temperature variations. Local climate data were acquired from an Australian Bureau of Meteorology station less than 50 km from the catch site. The research was conducted under scientific research permits issued by the Animal Ethics Committees at the University of Gothenburg, Sweden (protocol number: 001066) and at the University of Sydney, Australia (protocol number: 2013/6050).

### 2.2 Egg collection, incubation, and sexing of hatchlings

**Figure 1:**
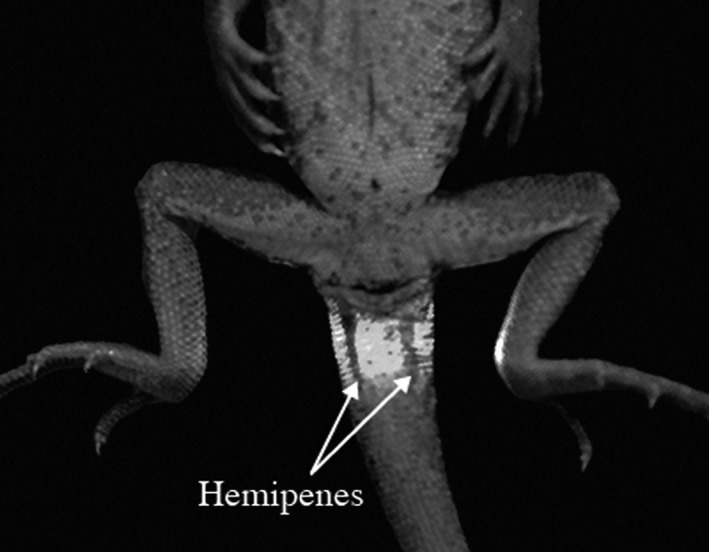
*Ctenophorus pictus* hatchling hemipenes revealed by hemipenal transillumination

Cages were checked daily for recently laid clutches. Females laid 1–4 clutches with an average of 1.63 ± 0.20 (mean ± *SE*,* N *=* *19) clutches with an average 3.67 ± 0.27 (mean ± *SE*,* N *=* *30) eggs per clutch. There were 19 first clutches (i.e. first clutch laid by a female), 8‐second clutches, three‐third clutches (Supporting information Table S1). Any eggs that were overtly unhealthy were discarded; the remaining were incubated (*N *=* *110 from 19 females). The true proportion of fertilized eggs was, however, unknown. Prior to incubation, the eggs were cleaned from sand and moisture, weighed to the nearest 0.001 g, and placed individually in 125‐ml disposable plastic cups half‐buried in moist vermiculite (1:5 by volume, resulting approximately a −12 kPa water potential) filling a third of the cup. The cups were sealed using plastic cling wrap and a rubber band to prevent extensive moisture loss and placed in incubators set at constant 28, 30, and 32°C. Treatment temperatures were chosen based on the previously suggested ideal incubation temperature of 30°C (Olsson et al., 2007). The actual temperature in each incubator was measured using HOBO H08 data loggers (Onset Computer Corporation, Bourne, MA, USA) throughout the entire experiment (191 days). The actual temperatures in the incubators were in the 28, 30, and 32°C treatment (mean °C ± *SE*): 27.9 ± 0.002 (*N *=* *13,337), 29.9 ± 0.004 (*N *=* *10,055), and 31.8 ± 0.004 (*N *=* *15,453), respectively. Eggs from a single clutch were allocated equally across the three temperature treatments, in concordance with a split‐clutch design. To minimize the effect of possible thermal gradients inside the incubators, the cups were rotated among three shelves in each incubator every 2 weeks. Water was added to each cup during this rotation accounting for the small, but clear, loss of water through evaporation (about 1 g per week). Incubators were checked daily for hatchlings and dead eggs. The sex of hatchlings was determined by hemipenal transillumination in males (Brown, 2009) after a few weeks, as hemepenes were easier to identify at this stage (Figure 1). Hatchlings that died prior to sexing were frozen for later sexing. Some deceased offspring, however, could unfortunately not be sexed due to the compromised tissue quality and lack of vascularization on which transillumination sexing depends.

### 2.3 Statistical analysis

The effect of incubation temperature on sex was analyzed using sex (binary data) as the dependent variable, mean incubation temperature as fixed factor, and parental identity as random effect. Maternal and paternal identity could unfortunately not be separated as females were housed with the same male throughout the experiment. The model was fitted following a generalized linear mixed model (GLMM) approach using the PROC GLIMMIX macro in SAS v.9.4 with a binomial distribution and a logit link function (Littell, Milliken, Stroup, & Wolfinger, 1996). Effect of temperature was tested using a *F* test and the random effect using a likelihood ratio test.

## 3 RESULTS

**Figure 2:**
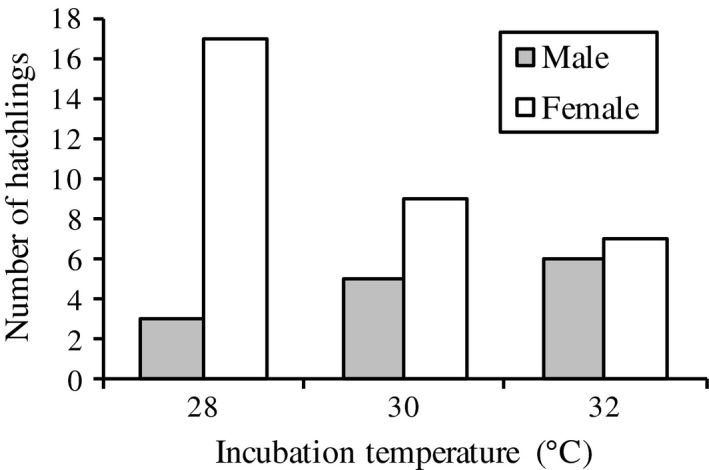
Number of male and female hatchlings among the three temperature treatments

**Figure 3:**
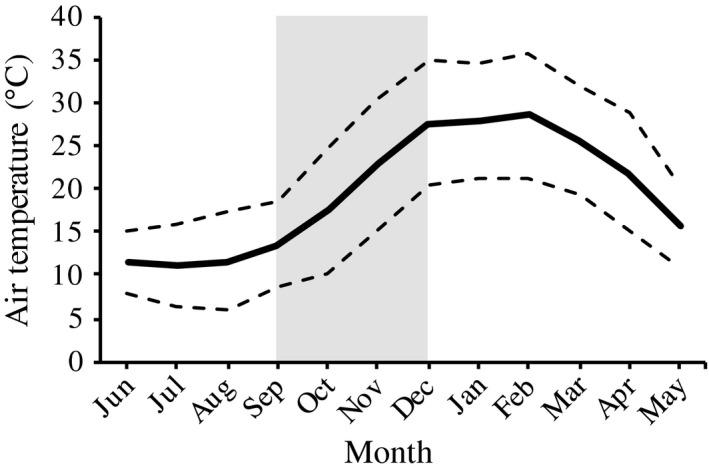
Mean monthly air temperatures for Cobar, Australia during 2016. The solid line represents mean temperature and dotted lines mean maximum and mean minimum temperature. Shaded area shows the natural breeding season of *Ctenophorus pictus*. Data acquired from the Australian Bureau of Meteorology

The sex ratios in the three temperature treatments were consistently female‐biased (proportion of males to total number of hatchlings ± *SE*): 0.15 ± 0.08 (28°C, *N *=* *20), 0.36 ± 0.13 (30°C, *N *=* *14) and 0.46 ± 0.13 (32°C, *N *=* *13), see Figure 2. Both incubation temperature and parental identity affected offspring sex (Table 1), with a higher proportion of females produced at lower temperatures. The mean monthly air temperature increased steadily throughout the natural breeding season (September to December), with an average increase of 4.7°C per month (Figure 3). The egg mortality was 41% (Supporting information Table S2).

**Table 1:** Results from the generalized linear mixed model, testing the effect of temperature (fixed effect) and parental identity (random effect) on hatchling sex

	Sex				
	Estimate	*N*	*df*	*χ* ^*2*^	*p*
Random effect					
Parental identity	1.309	47	1	156.38	<0.001^a^

	*ndf*	*ddf*	*F*	*p*
Fixed effect				
Incubation temperature	2	28	4.05	0.029^a^

a Significant value (*p *<* *0.05).

## 4 DISCUSSION

The research of reptiles has consistently shown that sex allocation cannot be explained with discrete sex determination systems (GSD or ESD) but requires a continuum between the two (Sarre et al., 2004), with a varying degree of influence from genetic and environmental factors depending on species. Here, we provide evidence for TSD in *C. pictus*, as predicted by the model proposed by Warner et al. (2009). We also show that parental identity affects offspring sex ratio, consistent with the findings of Uller et al. (2006). These results clearly show the complexity of the sex determination system in this species, and below, we discuss these results in turn.

### 4.1 Temperature effects

Experimental work exploring the effect of temperature on sex allocation in *C. pictus* has, inconsistently, shown both the presence (present study; Harlow, 2004) and absence of TSD (Uller et al., 2006). The former having somewhat small sample sizes, something not uncommon in incubation experiments, with an unpredictable number of fertilized clutches laid and hatching success. Albeit a larger sample size, Uller et al. (2006) did not follow a split‐clutch design, potentially obscuring possible temperature effects. This may be particularly true as the identity of parents has been shown to affect the sex ratio of offspring in this species (present study; Uller et al., 2006). The sex ratios from the different temperature treatments in Uller et al.'s study are all close to 40% males. In our study, however, the sex ratio increases with temperature, from very few males in the lowest temperature treatment to approaching 50% males in the highest temperature treatment. Furthermore, the model proposed by Warner et al. (2009) predicts that selection should favor a thermal effect on sex determination in *C. pictus*. The model expects that selection for TSD will be strongest in reptiles with: (a) a prolonged breeding season; (b) a short lifespan; (c) intense male‐male competition; and (d) little effect of hatching date (time of year) on the survival of juveniles. The three‐first factors are easily tested, and *C. pictus* possess all three. The fourth factor is more difficult to examine, and field data on short‐lived reptiles are limited. Hatching date was, however, found not to affect juvenile survival differently among the sexes in the Australian jacky dragon, *Amphibolurus muricatus* (Warner & Shine, 2005). In accordance with Warner et al.'s model, females produced at lower incubation temperature assume an increased probability of early reproductive success. Furthermore, the high male‐male competition over mating opportunities will most likely severely limit the early reproductive success of younger males when in contest with larger territorial males from previous cohorts (Aragón, López, & Martín, 2006; Jenssen, Decourcy, & Congdon, 2005; Olsson, 1992).

If such variation of reproductive success occurs in *C. pictus*, seasonal timing of hatching could be the outcome of: (a) active female nest‐site choice, resulting in eggs being laid in different nest‐sites early versus late in the season; or (b) passive nest‐site choice, resulting in eggs being exposed to seasonal differences in nest temperatures. In the first scenario, maternal nest‐site choice could shift seasonally, selecting nests with a temperature that would produce the sex most favored to that time of the season. Although the presence of nest‐site choice in *C. pictus* has not yet been investigated, a close relative (Hugall, Foster, Hutchinson, & Lee, 2008), the jacky dragon, chooses nest‐sites adaptively (Warner & Shine, 2008). In the second scenario, however, adaptive maternal nest‐site selection is not required but is simply consistent with seasonal changes in ambient (and thereby nest) temperatures. Seasonal thermal variations then act to produce the optimal sex linked to a particular season. In the case of this study, climate data showed that *C. pictus* experiences a seasonal increase in ambient temperatures during the breeding season, and in agreement with the model, the results showed that lower temperature (analogues to earlier in the season) produced a higher proportion of females. Although other studies on TSD in *Ctenophorus* spp. are relatively few, the genus seems to follow the “short‐lived agamid” model discussed above. Of the four species tested (*Ctenophorus decresii*,* Ctenophorus fordi*,* Ctenophorus ornatus* and *C. pictus*), all are short‐lived, have a prolonged breeding season, and male‐male competition over mating opportunities has been observed in all but *C. fordi*. In agreement with predictions, evidence for TSD has been found in all species but *C. fordi* (Harlow, 2000, 2004; Uller et al., 2008). In‐depth comparisons among the TSD species from past studies and the present study are difficult because previous work performed either has very small sample sizes in the higher thermal ranges or lack such treatments altogether. However, sample sizes are larger in the low temperature treatments and these show a similar result to the present study, with strongly female‐biased sex ratios.

A major limitation of this study is that we cannot rule out temperature‐by‐sex effects on mortality as an explanation for the observed differences in sex ratio among the tested incubation temperatures. This effect could be present during the egg stage leading to a greater mortality of one sex during embryogenesis and thus creating a false impression of TSD. An alternative explanation for the high proportion of unhatched eggs in this study is that they were never fertilized in the first place. In another study of the same species, they report on a hatching success consistently over 81% (Tobler, Healey, & Olsson, 2011), even when eggs were injected with testosterone. Thus, it is very unlikely that the current analysis is based on fully fertile males. As stated, females are only able to produce fertile eggs in the presence of males (Uller & Olsson, 2005) which of course assumes that the males mating with them are fully fertile. We believe that the artificial hibernation may produce variations in male fertility, some males may not yet have reached full fertility since “spring” emergence, as shown in other hibernating reptiles (Olsson & Madsen, 1996).

We found a significant temperature‐by‐sex effect on posthatching mortality (Supporting information Table S3) which is important as we were unable to sex about half of the hatchlings that died prior to sexing. If a similar effect was true in these unsexed hatchlings, this difference could explain the observed differences in sex ratio among the incubation temperatures. This limitation in the experimental design could easily have been avoided by sexing the hatchlings earlier and thus eliminating the posthatching mortality and tissue‐quality problem.

### 4.2 Parental effects

Parental effects on sex ratio has been found in *C. pictus* (present study; Uller et al., 2006), suggesting a sex determination system more complex than either one end of the GSD‐ESD continuum. A recent study by Warner, Uller, and Shine (2013) found novel evidence for a transgenerational effect on sex determination in the jacky dragon. They show that the incubation temperature experienced by the father affected the sex of the offspring, an effect sustained for several years. This recent discovery gives an additional level to an ever increasing complexity of sex determination in Australian agamid lizards. The jacky dragon and painted dragon share many life‐history traits, both are short‐lived, have a prolonged breeding season, are territorial, and have a temperature aspect to their sex determination (in agreement with the “short‐lived agamid” model discussed above). This transgenerational sex determination found in a closely related species to *C. pictus* offers interesting opportunities for further research. It is, however, more likely that the parental effect on sex ratio in this study is maternally derived, as previously shown by Uller et al. (2006), with a consistent effect of female identity on sex ratio. Although this study does not examine the source of this parental influence, our results clearly show that the painted dragon possesses a more complex sex determination system than previously thought. Furthermore, heritable variations for sex ratios in species with TSD have consistently been shown in reptiles (Bull, Vogt, & Bulmer, 1982; Janzen, 1992; Rhen & Lang, 1998; Rhen, Schroeder, Sakata, Huang, & Crews, 2011). It is likely that the parental effect on sex ratio found in this study is due to maternally heritable differences in sex determining reaction norms, effectively creating a variation between females or embryos in how the developmental system respond to temperature.

To date, research on *C. pictus* demonstrates an increasingly complex sex determination system, including effects of incubation temperature and possibly genetic and epigenetic factors (Harlow, 2004; Uller et al., 2006; Warner et al., 2013). Although cytogenetically distinct sex chromosomes have not been found in this species (Uller et al., 2006), more advanced high‐resolution molecular techniques, such as comparative genomic hybridization, may detect subtle differences in chromosomes between the sexes, as shown in the freshwater turtle *Chelodina longicollis* (Ezaz et al., 2006). The current repertoire of research on short‐lived agamid lizards seems to follow the “short‐lived agamid” model as an explanation for the adaptive significance and evolution of TSD in this group. Furthermore, the variation in sex determination systems among closely related species (Uller et al., 2006, 2008) and the presence of both environmental and genetic aspects to sex determination in Australian agamid lizards offer a novel opportunity to address fundamental questions on the evolution of sex determination systems.

## CONFLICT OF INTEREST

None declared.

## AUTHOR CONTRIBUTIONS

AH and MO conceived the ideas and designed methodology; AH collected the data; AH and MO analyzed the data; AH led the writing of the manuscript. All authors contributed critically to the drafts and gave final approval for publication.

## DATA ACCESSIBILITY

The dataset supporting this article are available in Dryad https://doi.org/10.5061/dryad.r6nq870.
