# Supplementary material for: Incubation temperature and parental identity determine sex in the Australian agamid lizard Ctenophorus pictus
Source: Ecol Evol. 2018 Sep 3;8(19):9827–33. doi: 10.1002/ece3.4466 (PMC6202699; doi:10.1002/ece3.4466)
Supplement: Supplementary file 1 [file ECE3-8-9827-s001.docx]

Supplementary table 1. Clutch data for all females.

|  |  | Number of eggs per clutch | | |
| --- | --- | --- | --- | --- |
| Female ID | Number of clutches | First | Second | Third |
| 3 | 1 | 3 | - | - |
| 6 | 1 | 7 | - | - |
| 7 | 1 | 3 | - | - |
| 10 | 3 | 1 | 2 | 5 |
| 13 | 1 | 5 | - | - |
| 16 | 3 | 4 | 5 | 5 |
| 18 | 2 | 5 | 2 | - |
| 21 | 1 | 2 | - | - |
| 22 | 3 | 4 | 5 | 5 |
| 23 | 1 | 5 | - | - |
| 28 | 2 | 3 | 5 | - |
| 32 | 1 | 3 | - | - |
| 33 | 1 | 3 | - | - |
| 36 | 1 | 3 | - | - |
| 47 | 2 | 2 | 5 | - |
| 48 | 1 | 1 | - | - |
| 52 | 2 | 4 | 4 | - |
| 53 | 1 | 4 | - | - |
| 57 | 2 | 1 | 4 | - |

Supplementary table 2. Mortality data of eggs and hatchlings.

|  | Incubation temperature (°C) | | |
| --- | --- | --- | --- |
| Number | 28 | 30 | 32 |
| Unhatched eggs | 12 | 16 | 17 |
| Unsexed dead hatchlings | 4 | 6 | 8 |
| Dead female hatchlings | 2 | 6 | 5 |
| Dead male hatchlings | 2 | 1 | 1 |
| Surviving female hatchlings | 15 | 3 | 2 |
| Surviving male hatchlings | 1 | 4 | 5 |

Supplementary table 3. Post-hatching mortality statistics (*N*=47) of the effect of temperature treatment and sex on post-hatching morality. The models were fitted in R (R Core Team 2016: version 3.3.2) following a generalized linear mixed model (GLMM) and a linear mixed model (LMM) approach using the *glmer* and *lmer* function from the lme4 package (Bates et al. 2015) with paternal identity as random effect. Estimates of *P*-values were obtained using parametric likelihood ratio bootstrapping with the *PBmodcomp* function from the pbkrtest. package (Ulrich and Søren 2014).

|  | Post-hatching mortality | | |
| --- | --- | --- | --- |
|  | df | *χ^2^* | *P* |
| Incubation temperature | 1 | 1.87 | 0.21 |
| Sex | 1 | 2.55 | 0.13 |
| Incubation temperature $\times$ Sex | 1 | 15.01 | <0.001 |

R Core Team, 2016. R: A language and environment for statistical computing. R Foundation, Austria.

Bates, D.M., M. Machler, B.M. Bolker, and S.C. Walker. 2015. Fitting linear mixed-effects models using lme4. Journal of Statistical Software, 67: 1-48.

Ulrich, H., and H. Søren. 2014. A Kenward-Roger approximation and parametric bootstrap methods for tests in linear mixed models - the R package pbkrtest. Journal of Statistical Software, 59: 1-30.
